# A Treatise on Nervous Diseases: Their Symptoms and Treatment

**Published:** 1885-11

**Authors:** 


**A Treatise on Nervous Diseases: Their Symptoms and Treatment.** A text-book for students and practitioners, by SAMUEL G. WEBBER, M.D., Clinical Instructor in Nervous Diseases, Harvard Medical School; Visiting Physician for Diseases of the Nervous System at the Boston City Hospital, etc. New York: D. Appleton & Co. 1885.

After carefully reading this work, we certainly hope that this work will be used as a "text-book for students" and the general practitioner. With this book as their guide, students will not be led too soon into indecision concerning doubtful subjects which are so frequently discussed and reiterated in so many works on nervous diseases. While we would not say it should take the place of such a work as Ross', it will prove a valuable assistant to the better understanding of such a work. The first chapter is on the "Methods of examining sensation and motion in patients affected with nervous diseases." The few pages devoted to this subject cover it completely, and leave us to wonder how such a vast subject is condensed into this small space. Chapter two is on "Anatomy, physiology and general symptomatology of the diseases of the brain," and what is said of the first is quite true of this. The chapters on Cerebral Anæmia, Hyperæmia and Neurasthenia are especially interesting and instructive. Thus we might analyze each part of this work and find little to condemn and much to praise in it. We close with the hope that this work *will* become a text-book for every student, and will find its way into the hands of many practitioners and specialists in this branch.

